# Supplementary material for: One-Stage Synovectomies Result in Improved Short-Term Outcomes Compared to Two-Stage Synovectomies of Diffuse-Type Tenosynovial Giant Cell Tumor (D-TGCT) of the Knee: A Multicenter, Retrospective, Cohort Study
Source: Cancers (Basel). 2023 Feb 2;15(3):941. doi: 10.3390/cancers15030941 (PMC9913566; doi:10.3390/cancers15030941)
Supplement: Supplementary file 1 [file cancers-15-00941-s001.zip › cancers-2162022-supplementary.pdf]

Supplementary table s1. One- vs two-stage synovectomies per center.

Centers	One-stage	Two-stage
LUMC	20	19
Previous treatment (%)	13 (59)	8 (42)
Median Follow-up, months (range)	18 (10-123)	62 (3-194)
RUMC	17	19
Previous treatment (%)	11 (58)	11 (58)
Median Follow-up, months (range)	86 (6-200)	95 (9-199)
MSH	31	
Previous treatment (%)	18 (58)	-
Median Follow-up, months (range)	57 (12-157)	
AUMC	6	17
Previous treatment (%)	2 (33)	9 (53)
Median Follow-up, months (range)	11 (2-62)	39 (4-203)
MAYO	11	6
Previous treatment (%)	8 (73)	4 (67)
Median Follow-up, months (range)	45 (10-137)	32 (6-52)
MCW	15	
Previous treatment (%)	3 (20)	-
Median Follow-up, months (range)	54 (4-84)	
RPAH		12
Previous treatment (%)	-	1 (8)
Median Follow-up, months (range)		62 (4-104)
UCD	10	
Previous treatment (%)	4 (40)	-
Median Follow-up, months (range)	16 (1-35)	
UCLA	7	1
Previous treatment (%)	4 (57)	(100)
Median Follow-up, months (range)	49 (23-159)	58
Total	117	74
Previous treatment (%)	59 (50)	34 (46)
Median Follow-up, months (range)	45 (1-200)	59 (3-203)

Supplementary table s2. Outcomes for open one- and two-stage synovectomies in particular.

Features	One-stage synovectomy open N = 58	Two-stage synovectomy open N = 67	P-value
Length of hospital stay, days, median (range)[b]	N=56<br>6 (1-13)	N=63<br>6 (3-26)	0.008
Maximum range of motion, degrees, median (range)	N=51	N=46	
Flexion	125 (70-145)	120 (45-140)	0.126
Extension	0 (0-20†)	0 (0-10†)	0.253
Complications	N=58	N=64	
Yes	13 (22%)	23 (36%)	0.069
No	45 (78%)	41 (64%)	
Radiological progression	N=57	N=66	
Yes	23 (40%)	31 (47%)	0.371
No	34 (60%)	35 (53%)	

†The number of degrees equals the degrees of extension lag

Supplementary table s3. Outcomes per different techniques performed for one-stage synovectomies.

Features	Complete open approach N=58	Combined approach N=51	Complete arthroscopic approach N=14	P-value
Length of hospital stay, days, median (range)[b]	N=56<br>6 (1-13)	N=53<br>3 (1-8)	N=53<br>1 (1-4)	<0.0001
Maximum range of motion, degrees, median (range)	N=51	N=46	N=42	
Flexion	125 (70-145)	120 (90-140)	127.5 (90-140)	0.654
Extension	0 (0-20†)	3 (0-30†)	0 (0-15†)	0.092
Complications	N=58	N=50	N=12	
Yes	13 (22%)	14 (28%)	-	0.009
No	45 (78%)	36 (72%)	12 (100%)	
Radiological progression	N=57	N=51	N=12	
Yes	23 (40%)	17 (33%)	5 (42%)	0.069
No	34 (60%)	34 (67%)	7 (58%)	

†The number of degrees equals the degrees of extension lag

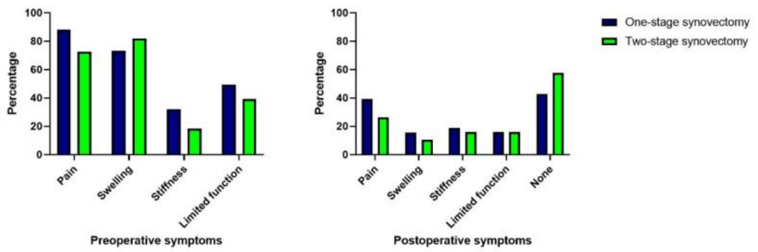

Supplementary figure s1. Pre- and postoperative symptoms of patients undergoing a one- or two-stage synovectomy and no subsequent treatment

Formatted: Automatically adjust right indent when grid is defined, Snap to grid

Commented [SG(1]: These tables are duplicate and should be removed

Supplementary table 2. Outcomes for open one- and two-stage synovectomies in particular.

Features	One-stage synovectomy open N = 58	Two-stage synovectomy open N = 67	P-value
Length of hospital stay, days, median (range)[b]	N=56<br>6 (1-13)	N=63<br>6 (3-26)	0.008
Maximum range of motion, degrees, median (range)	N=51	N=46	
Flexion	125 (70-145)	120 (45-140)	0.126
Extension	0 (0-20†)	0 (0-10†)	0.253
Complications	N=58	N=64	
Yes	13 (22%)	23 (36%)	0.069
No	45 (78%)	41 (64%)	
Radiological progression	N=57	N=66	
Yes	23 (40%)	31 (47%)	0.371
No	34 (60%)	35 (53%)	

Features	One-stage synovectomy open N = 58	Two-stage synovectomy open N = 67	P-value
Length of hospital stay, days, median (range)[b]	N=56<br>6 (1-13)	N=63<br>6 (3-26)	0.008
Maximum range of motion, degrees, median (range)	N=51	N=46	
Flexion	125 (70-145)	120 (45-140)	0.126
Extension	0 (0-20†)	0 (0-10†)	0.253
